# Eutectic Phenomenon of LiNH_2_-KH Composite in MH-NH_3_ Hydrogen Storage System

**DOI:** 10.3390/molecules24071348

**Published:** 2019-04-05

**Authors:** Kiyotaka Goshome, Ankur Jain, Hiroki Miyaoka, Hikaru Yamamoto, Yoshitsugu Kojima, Takayuki Ichikawa

**Affiliations:** 1 Renewable Energy Research Center, National Institute of Advanced Industrial Science and Technology, 2-2-9 Machiikedai, Koriyama, Fukushima 963-0298, Japan; goshoume.kiyotaka@aist.go.jp; 2 Natural Science Center for Basic Research and Development, Hiroshima University, 1-3-1 Kagamiyama, Higashi-Hiroshima 739-8530, Japan; miyaoka@h2.hiroshima-u.ac.jp (H.M.); mhikaru@h2.hiroshima-u.ac.jp (H.Y.); kojimay@hiroshima-u.ac.jp (Y.K.); 3 Graduate School of Advanced Sciences of Matter, Hiroshima University, 1-3-1 Kagamiyama, Higashi-Hiroshima 739-8530, Japan; 4 Graduate School of Engineering, Hiroshima University, 1-4-1 Kagamiyama, Higashi-Hiroshima 739-8527, Japan

**Keywords:** hydrogen carrier, ammonia, metal hydride, double-cation amide, eutectic melting

## Abstract

Hydrogenation of a lithium-potassium (double-cation) amide (LiK(NH_2_)_2_), which is generated as a product by ammonolysis of litium hydride and potassium hydride (LiH-KH) composite, is investigated in details. As a result, lithium amide (LiNH_2_) and KH are generated after hydrogenation at 160 °C as an intermediate. It is noteworthy that the mixture of LiH and KNH_2_ has a much lower melting point than that of the individual melting points of LiNH_2_ and KH, which is recognized as a eutectic phenomenon. The hydrogenation temperature of LiNH_2_ in the mixture is found to be significantly lower than that of LiNH_2_ itself. This improvement of reactivity must be due to kinetic modification, induced by the enhanced atomic mobility due to the eutectic interaction.

## 1. Introduction

The use of hydrogen energy has attracted attention for the introduction of renewable energy [1]. Since renewable energy fluctuates significantly depending on the season, weather, and location, it is necessary to stably store and transport it by conversion to a suitable chemical energy. Hydrogen can be produced by water electrolysis using renewable energy-based power sources, and stored compactly at low pressure by using a hydrogen storage alloy [2]. Finally, hydrogen can be used with a fuel cell to obtain electric and heat power according to demand [3]. In terms of hydrogen transportation, gravimetric hydrogen density of hydrogen storage alloys are generally lower than 2 wt%, which is insufficient for transportation application [4]. Compressed and liquid hydrogen are the options; however, there are some problems to be solved for practical use (e.g., development of a lightweight pressure-tight tank, a durable hydrogen compressor, a cryogenic insulation container, a cryogenic liquid pump, and so on) [5]. Hydrides composed of light elements (e.g., LiH, NaBH_4_, CH_4_, BH_3_NH_3_, NH_3_, LiNH_2_, MgH_2_, and NaAlH_4_) are expected to be promising hydrogen storage materials, which can store hydrogen with high gravimetric and volumetric hydrogen density at moderate conditions [5,6,7,8]. Numerous studies for hydrogen storage properties of the hydrides and their combinations have been reported so far. The combination of solid hydrides (i.e., LiH and LiNH_2_) is one of the most notable systems and is known as an amide-imide (*M*-N-H) hydrogen storage system [9,10,11,12,13,14,15]. The first report of the above system was published by Chen et al. in 2002 [16]. The hydrogen absorption and desorption reaction with the high hydrogen capacity of 6.5 mass% proceeds as follows,

LiH + LiNH_2_ ↔ Li_2_NH + H_2_ (1)

The enthalpy change of the hydrogen release (ΔH_des_) by reaction (1) has been reported to be 65.6 kJ mol^−1^ H_2_ [17]. The equilibrium temperature was estimated to be 234 °C to obtain 1 atm of equilibrium hydrogen pressure when the entropy change was assumed as 130 J mol^−1^ K^−1^ [18]. To modify the thermodynamics, other *M*-N-H systems with alternative metals (e.g., K, Na, Ca, and Mg) have been studied so far. Besides, many researchers have studied these systems to reveal the reaction mechanism. Ichikawa et al. have clarified that the de/re-hydrogenation reaction is composed of two-step reactions, including the decomposition of LiNH_2_ to generate NH_3_ as follows [19],

2LiNH_2_ ↔ Li_2_NH + NH_3_ (2)

LiH+NH_3_ ↔ LiNH_2_ + H_2_ (3)

Improving the kinetics of the de/re-hydrogenation, and suppressing the emission of NH_3_ gas by reaction (2), could be achieved by using various catalysts, such as KOH, TiCl_3_, LiTi_2_O_4_, CeF_4_, etc. [15].

The metal hydride-ammonia (*M*H-NH_3_) hydrogen storage system was derived from the reaction in the equation (3). By using this system, H_2_ can be desorbed and absorbed below 300 °C [20,21,22,23,24]. Here, NH_3_ has a top-class hydrogen capacity; the gravimetric density is 17.8 mass%, and it condenses at 25 °C when pressurized to 1 MPa. Therefore, the *M*H-NH_3_ system is also regarded as a high gravimetric and volumetric hydrogen carrier (*M* = Li; 8.1 wt%, 4.5 kg /100 L) [21]. Previously, it was experimentally revealed that the exothermic ammonolysis of *M*H to generate H_2_ proceeds at room temperature [21,22,25,26,27,28,29]. Besides, the hydrogenation of *M*NH_2_ to form *M*H and NH_3_ can proceed below 300 °C. The reaction rate of both ammonolysis and hydrogenation become higher in the order of elements placed in the periodic table (K > Na > Li) [22]. Therefore, the kinetics of the LiH-NH_3_ system with the highest gravimetric hydrogen density needs to be improved. In previous work, we investigated the LiH-KH composite NH_3_ system [30]. The reaction rate of the ammonolysis of the LiH-KH composite was found to be improved by a synergetic effect. Here, a new double-cation metal amide phase (i.e., LiK(NH_2_)_2_) was reported to be formed as a reaction product. Interestingly, the complete hydrogenation of complex LiK(NH_2_)_2_ proceeded at a much lower temperature than that of LiNH_2_. In this work, thermal and hydrogenation properties of complex LiK(NH_2_)_2_ are investigated in further detail to understand its reaction mechanism, which would be useful and applicable to some related *M*-N-H systems.

## 2. Results and Discussion

### 2.1. Investigation for Hydrogenation of Complex LiK(NH_2_)_2_ at Lower Temperature

The hydrogenation of amides (*M*(NH_2_)*_m_*) proceeds by the following reaction,

*M*(NH_2_)*_m_* + *m*H_2_ → *M*H*_m_* + *m*NH_3_ (4)

where *m* represents the stoichiometric coefficient and is equal to the valence number of the metal cation (*M^m+^*). The hydrogenation reaction proceeds through an ion-exchange reaction between solid *M*(NH_2_)*_m_* and gaseous H_2_, that is, amide ions (NH_2_^−^) in *M*(NH_2_)*_m_* are replaced by hydride ions (H^−^) to form solid *M*H*_m_* and gaseous NH_3_. Thus, the weight loss during the hydrogenation is shown due to the weight difference between *M*(NH_2_)*_m_* and *M*H*_m_*. The reaction yield (*Y*) is estimated by the weight loss of the solid samples after the hydrogenation by the following equation,

*Y* = 100⋅|Δ*w*|⋅*W_M_*_(NH2)*m*_ / (*m*⋅*w*⋅*W*_NH_) (5)

where *w*, Δ*w*, *W_M_*_(NH2)*m*_, and *W*_NH_ represent the weight of the sample before the reaction, the weight loss of the sample due to the hydrogenation, the molecular weight of *M*(NH_2_)*_m_*, and NH, respectively. The hydrogenation of complex LiK(NH_2_)_2_ was performed under hydrogen flow condition at adequate flow rate as compared to a small amount of sample; therefore, the generated NH_3_ was removed continuously from the reaction field to maintain the non-equilibrium condition during the reaction [31]. Thus, the endothermic hydrogenation could proceed even below 300 °C.

**Figure 1:**
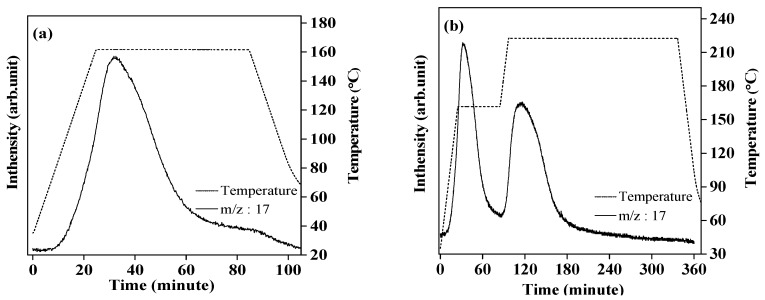
Thermal desorption mass spectroscopy (TD-MS) profile of complex LiK(NH_2_)_2_ under 0.5 MPa H_2_ flow condition at 160 °C for 1 h (**a**), and following 220 °C for 4 h (**b**), where the heating rate is 5 °C/min and m/z is 17 for NH_3_.

Figure 1a shows the thermal desorption mass spectroscopy (TD-MS) profile of complex LiK(NH_2_)_2_ under 0.5MPa H_2_ flow condition during heating process to 160 °C and the isothermal step for 1 h. In the TD-MS profile, a peak corresponding to NH_3_ was originated at 80 °C, which decreased during the isothermal step, suggesting that the hydrogenation by the reaction described in Equation (4) proceeded to form NH_3_. The reaction yield was only 56.3%, which means that half the amount of metal amides remained unreacted.

**Figure 2:**
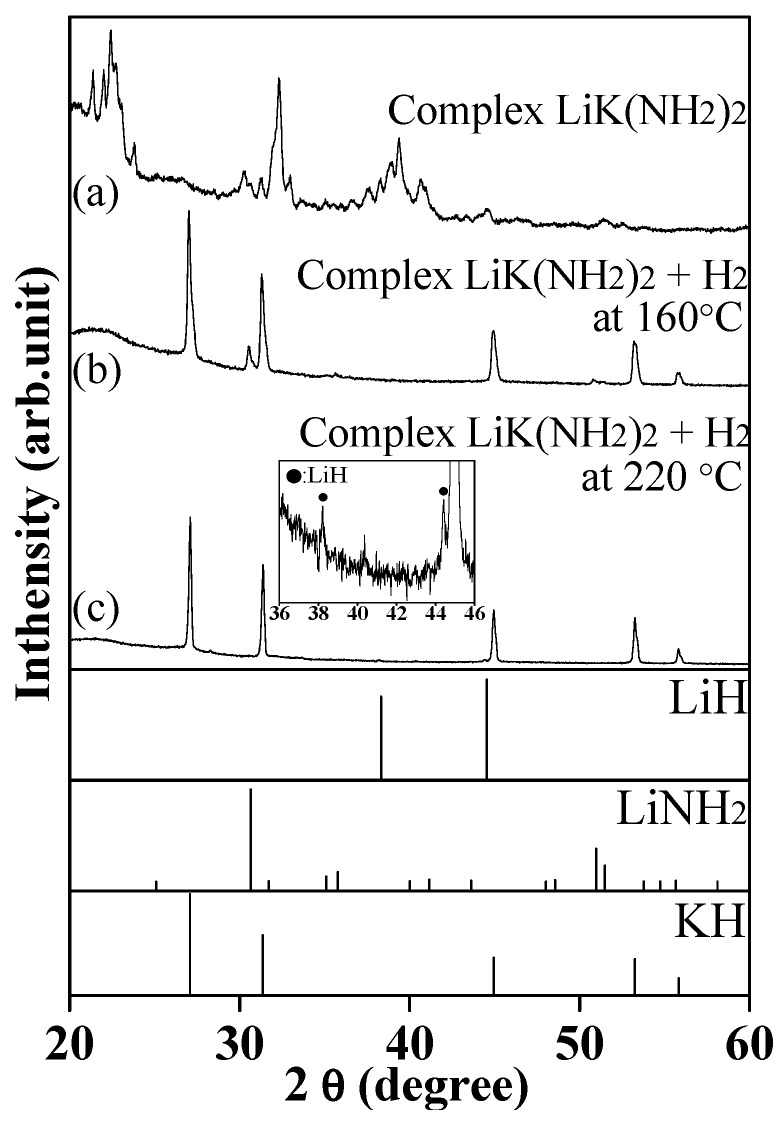
X-ray diffraction (XRD) patterns of complex LiK(NH_2_)_2_ before (**a**) and after (**b**) the hydrogenation at 160 °C for 1 h, and following 220 °C for 4 h (**c**). The inset shows the enlarged figure for profile (**c**) to find the peaks of LiH; XRD pattern of LiH (PDF#78-0838), LiNH_2_ (PDF#75-0049), and KH (PDF#65-9244) are referred from databases.

Figure 2a,b show X-ray diffraction (XRD) patterns of the samples before and after the hydrogenation at 160 °C for 1 h. The peaks of complex LiK(NH_2_)_2_ phase completely disappeared, and the diffraction patterns corresponded to the KH and LiNH_2_ phases. It was considered that KH and LiNH_2_ were formed by the reaction of complex LiK(NH_2_)_2_ with H_2_ below 160 °C, in other words, the K–related components in the complex amide selectively reacted with H_2_ prior to the reaction of the Li-related component. The phase variation at 160 °C was consistent with the above approximately 50% reaction yield, estimated from the weight change, because the atomic ratio of Li:K in the complex LiK(NH_2_)_2_ was 1. Here, the slightly excess reaction yield, beyond 50%, must be caused by the partial hydrogenation of the LiNH_2_ component; however, the diffraction peaks corresponding to LiH could not be detected by XRD because of the small amount and low diffraction intensity, which was due to the low electron number. The above consideration about the reactivity of KNH_2_ and LiNH_2_ components was consistent with the previous reports. The complex LiK(NH_2_)_2_ was hydrogenated to form a mixture of LiNH_2_ and KH through the formation of LiK_3_(NH_2_)_4_ as an intermediate [30]. Besides, the hydrogenation of KNH_2_ under H_2_ flow condition proceeded even at 50 °C, which was significantly lower than that of LiNH_2_ [22]. The above results indicate that the molecular properties of KNH_2_ and LiNH_2_ remained intact in LiK(NH_2_)_2_ and strongly affected the hydrogenation properties, even though crystal structure of the complex amide was different from those of the single cation amides.

In order to understand the thermal properties of the hydrogenated product of LiK(NH_2_)_2_ at 160 °C (i.e., the mixture of LiNH_2_ and KH), the differential scanning calorimetery (DSC) measurement under 0.5 MPa H_2_ atmosphere in a closed condition was performed, where the H_2_ atmosphere prevents the H_2_ desorption from the mixture of LiNH_2_ and KH by the equivalent process, described as Equation (1). A reversible sharp peak around 240 °C was observed in the DSC profile (Figure S1). Since no phase transition is known for either LiNH_2_ or KH at this temperature, this reversible peak suggested a possibility of melting; however, the melting point of both of LiNH_2_ and KH are also higher than 350 °C [22,32]. Therefore, the phase transition must have originated in the coexistence of KH and LiNH_2_, indicating a eutectic melting phenomenon. To further understand the reversible change of the mixture during the DSC measurement, and to confirm whether it is melting or not, the thermal behavior of a ball-milled mixture of KH and LiNH_2_ (similar to the hydrogenation product of complex LiK(NH_2_)_2_) was investigated by the DSC measurements.

**Figure 3:**
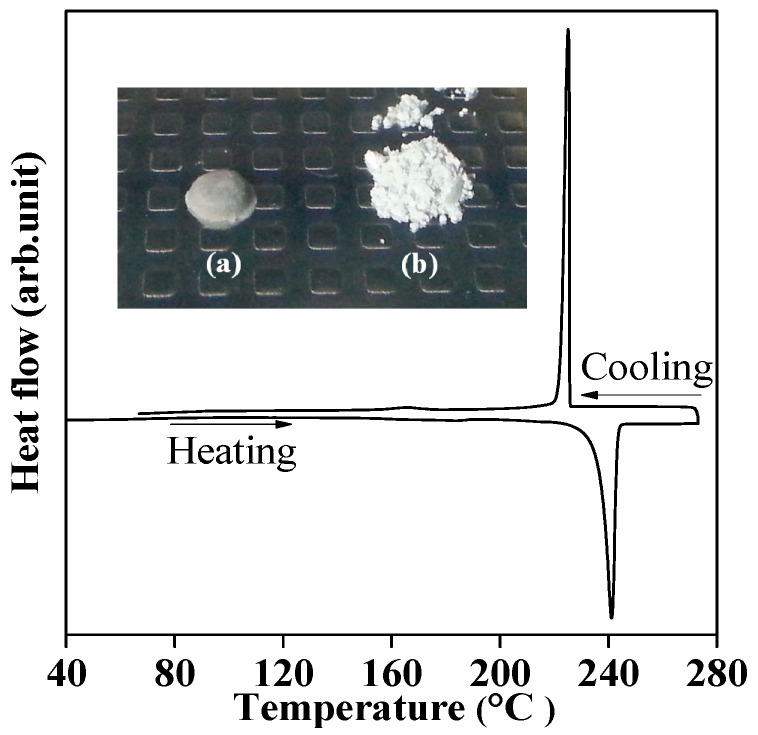
Differential scanning calorimetery (DSC) profile of the ball-milled mixture of LiNH_2_ and KH. The DSC was performed under 0.5 MPa H_2_ atmosphere in closed condition up to 270 °C, with heating rate of 5 °C/min. The inset shows the appearance of the ball-milled mixture of KH and LiNH_2_ before (**b**) and after (**a**) the DSC measurement.

**Figure 4:**
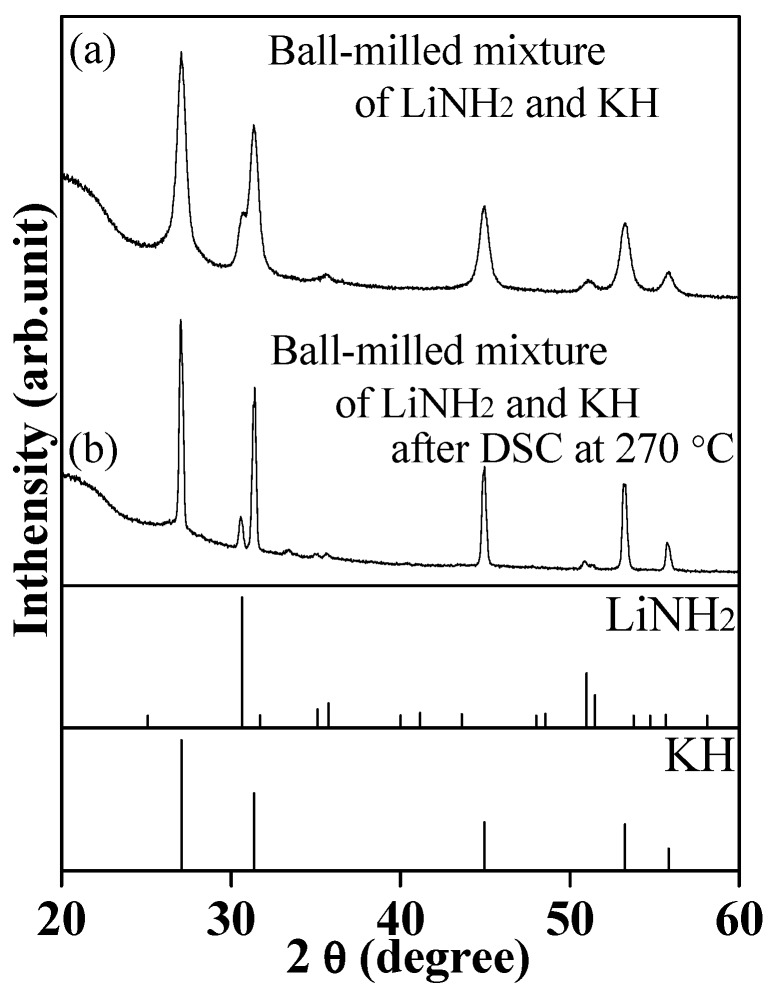
XRD patterns of the ball-milled mixture of LiNH_2_ and KH before (**a**) and after (**b**) DSC measurement under H_2_ atmosphere up to 270 °C with a heating rate of 5 °C/min; XRD patterns of LiNH_2_ (PDF#75-0049) and KH (PDF#65-9244) are referred from databases.

Figure 3 shows the DSC profile for the ball-milled mixture of KH and LiNH_2_ in the temperature range from room temperature (RT, 22 °C) to 270 °C under 0.5 MPa H_2_ atmosphere in the closed condition. The ball-milled mixture of KH and LiNH_2_ showed a similar reversible sharp peak around 240 °C. It is noteworthy that the temperatures of these peaks were not shifted even under the different H_2_ pressures (Figure S2), indicating that the reversible peaks were not originated due to de/re-hydrogenation. Besides, the sample after the DSC measurement was in a lump state, as shown in the insert image of Figure 3. Moreover, the XRD results of the sample before and after DSC measurement confirmed the existence of LiNH_2_ and KH phases, as shown in Figure 4. Thus, it was experimentally revealed that a mixture of LiNH_2_ and KH shows the eutectic phenomenon. Herein, the composition of the mixture of LiNH_2_/KH is not necessarily in a eutectic composition and might show a suspension after the melting. The accurate eutectic composition will be revealed by performing DSC measurements for the mixture of various compositions in the future.

### 2.2. Investigation for Hydrogenation of Complex LiK(NH_2_)_2_ at Higher Temperature

Further hydrogenation of the mixture of LiNH_2_ and KH, produced from the partial hydrogenation at 160 °C, was performed at 220 °C, which is lower than that of the eutectic melting point to avoid the influence of the phase transition, such as the decrease in surface area by melting. Figure 1b shows TD-MS profile of complex LiK(NH_2_)_2_ under 0.5 MPa H_2_ flow condition up to 220 °C; the temperature was increased and kept at 160 °C for 1 h and 220 °C for 4 h during the DSC scan. The intensity of the MS peak corresponding to NH_3_ obviously increased above 180 °C after the first reaction at 160 °C, which should be attributed to the hydrogenation of the residual LiNH_2_. In fact, XRD results in Figure 2c showed the diffraction pattern of KH without that of LiNH_2_. The existence of LiH was also confirmed in the enlarged XRD profile between 36 to 46 of 2θ range. The total reaction yield was found to be 95.6% and indicated the complete hydrogenation of complex LiK(NH_2_)_2_. In this view, the reaction yield in this work was much higher than the previous reports. Yamamoto et al. reported that the reaction yield for hydrogenation of single LiNH_2_ was 4.2% at 200 °C for 4 h and 71.0% at 300 °C [22]. Dong et al. reported that the reaction yield for hydrogenation of single LiNH_2_ was 24.3% at 200 °C for 4 h [24]. Even though it is difficult to directly compare our results with these previous reports because of the difference in H_2_ flow rate, reaction scale, and any other reaction conditions, it can be concluded that the hydrogenation performance of complex LiK(NH_2_)_2_ was obviously improved as compared with the LiNH_2_ itself, because it showed almost 100% hydrogenation at a relatively lower temperature and shorter retention time for the reaction. Furthermore, it can be suggested that the high reactivity of LiNH_2_ in the mixture at 220 °C originated from the eutectic interaction between KH and LiNH_2_. As a similar phenomenon, Yamamoto et al. [22] reported a sudden change in the hydrogenation of NaNH_2_ at around 200 °C; the temperature was quite close to the melting point of NaNH_2_ at 210 °C [33]. As described above, the melting point of LiNH_2_ was lowered down to about 240 °C by the eutectic interaction with KH. Particularly, the atoms in the solid phase possibly became active with high mobility near the melting point, resulting in high reactivity with H_2_ to form NH_3_. Such atomic mobility is important for the hydrogenation of complex LiK(NH_2_)_2_, because it is necessary to release heavy N atoms in the form of NH_3_. 

A lot of studies for *M*-N-H systems with various combinations of *M*_I_(NH_2_)*_m_*-*M*_II_H*_n_* composites (*M*_I_, *M*_II_ = Li, Na, K, Ca, Mg with the valence number of *M*_I_ and *M*_II_ cations as *m* and *n*) have been published before [34,35,36,37,38,39,40,41,42]. To cite an instance, an interaction between Mg(NH_2_)_2_ and LiH, known as Li-Mg-N-H system, has been regarded as one of the most practicable hydrogen storage material, because it can generate 5.5 mass% of H_2_ and be re-hydrogenated under 150 °C by the following reaction [43,44,45],

Mg(NH_2_)_2_ + 2LiH ↔ MgLi_2_(NH)_2_ + 2H_2_ (6)

Although the Li-Mg-N-H system has suitable thermodynamics (i.e., ΔH_des_ of −38.9 kJ mol^−1^ H_2_) and is expected to show desorption pressure of 0.1 MPa at <90 °C, its slow reaction rate is still the main issue for its practical use [46]. Thus, many approaches to improve the de/re-hydrogenation kinetics of the Li-Mg-N-H system have been reported [47,48,49,50,51]. It is noted that the eutectic melting phenomenon of *M*_I_(NH_2_)*_m_*-*M*_II_H*_n_* would be important as an advantage for such *M*-N-H system. As described above, the de-hydrogenation of the *M*-N-H system proceeds in the solid–solid reaction; therefore, the contact between the particles should be an important factor. Besides, it is reported that the de-hydrogenation process includes a decomposition process of metal amide to generate NH_3_ as an intermediate, then the decomposition could directly affect the de-hydrogenation rate. In this view, Leng et al. reported the decomposition properties of several metal amides [35]. Using the thermogravimetry-differential thermal analysis (TG-DTA), they have shown the two-step decomposition of LiNH_2_ at different temperatures. They claimed that these steps are originated in the decomposition on the surface at a lower temperature and in the bulk at higher temperature. Interestingly, the decomposition of the second step is likely to occur near the melting point of LiNH_2_ and due to the modification of the atomic mobility in the solid by the melting. From the above considerations of the contact between the particles and the decomposition rate of metal amide in the de-hydrogenation process of the *M*-N-H system, the melting phenomenon of the *M*_I_(NH_2_)*_m_*-*M*_II_H*_n_* composite should have a significant effect on the reaction kinetics. However, the melting phenomenon of the *M*_I_(NH_2_)*_m_*-*M*_II_H*_n_* composite has not been studied so far. In this work, the eutectic melting phenomenon of the mixture of KH and LiNH_2_ has been revealed for the first time by performing the thermal investigation in an unusual circumstance, that is a compressed H_2_ environment. Recently, Dong et al. reported that dehydrogenation properties of the Li-N-H system was improved by doping 1–10 mol% KH [52]; the eutectic phenomenon could be believed to be one of the factors for the improvement. Thus, a systematic detailed investigation of such eutectic melting phenomena is expected to lead to a breakthrough for the development of a novel *M*-N-H system in future.

## 3. Materials and Methods

Lithium hydride (LiH) (99.4%, Alfa aesar), potassium hydride (KH) (dispersion in mineral oil, Aldrich), lithium amide (LiNH_2_) (95%, Aldrich), and potassium amide (KNH_2_), synthesized by the reaction of KH with NH_3_ (99.999%), were used for the experiments. The KH with mineral oil was introduced into anhydrous tetrahydrofuran (THF) (>99.9%, Aldrich) and stirred for 3 min. Then, the supernatant solution was drained to remove the mineral oil. The above procedure was repeated 5 times. Finally, the slurry of the KH was evacuated for 5 h to completely remove the remaining THF. The complex LiK(NH_2_)_2_ was prepared by the milling of LiH and KH with a 1:1 molar ratio in a stainless steel container with twenty 7-mm steel balls, at 370 rpm for 10 h under 1.0 MPa H_2_ atmosphere, by using a planetary ball mill apparatus (Fritsch, P7). Then, this milled LiH-KH composite was heat-treated at 220 °C under 1.0 MPa H_2_ atmosphere and reacted with 0.8 MPa NH_3_ at room temperature to form complex LiK(NH_2_)_2_ [30]. A ball-milled mixture of LiNH_2_ and KH, with a molar ratio of LiNH2/KH = 1, was prepared by ball-milling for 10 h under 0.1 MPa Ar atmosphere. The samples were examined by X-ray diffraction (XRD) (Rigaku, RINT-2100) using Cu Kα radiation. The samples were sensitive to air and handled in a glove box (Miwa MFG, MP-P60 W) filled with highly pure Ar (>99.9999%).

Investigation for hydrogenation of complex LiK(NH_2_)_2_ was performed by thermal desorption mass spectroscopy (TD-MS) (Canon Anelva Corporation, M-100QA) (Kawasaki, Japan) under 0.5 MPa H_2_ flow condition at 200 sccm (= cc/min at 0.1 MPa, 0 °C), where 5 mg and/or less amount of sample was used. The weight of the sample before and after the TD-MS measurement was measured to estimate the reaction yield. The products, after the TD-MS measurements, were identified by XRD measurement.

Thermal properties of the mixture of LiNH_2_ and KH were investigated under 0.5–2 MPa H_2_ atmosphere by differential scanning calorimetry (DSC) (TA Instruments, Q10 PDSC). The products after the DSC measurements were identified by XRD measurement.

## 4. Conclusions

The hydrogenation reaction of complex LiK(NH_2_)_2_ is found to occur at 220 °C under H_2_ flow condition completely, which is lower than that of LiNH_2_. The hydrogenation was proceeded by two step reactions. At the first step, the hydrogenation of the KNH_2_ component in the complex amide occurred to form KH and NH_3_ below 160 °C, followed by the hydrogenation of LiNH_2_ to form LiH and NH_3_ at 220 °C. In this case, the reactivity of LiNH_2_ could be enhanced by the eutectic interaction with KH. Thus, it has been clarified that not only ammonolysis but also the hydrogenation of the LiH-NH_3_ system can be improved by the interaction with KH. In the future, the eutectic melting phenomena of the *M*_I_(NH_2_)*_m_*-*M*_II_H*_n_* composite will be investigated in further detail.

## Supplementary Materials

The following are available online, Figure S1: DSC profile of the reaction product after the hydrogenation of complex LiK(NH_2_)_2_ at 160 °C. The DSC was performed under 0.5 MPa H_2_ atmosphere in closed condition by heating up to 270 °C at 5 °C/min heating rate. Figure S2: DSC profile of the ball-milled mixture of LiNH_2_ and KH. The DSC was performed under 2.0 MPa H_2_ atmosphere in closed condition by heating up to 270 °C at 5 °C/min heating rate.
