# Supplementary material for: Eutectic Phenomenon of LiNH2-KH Composite in MH-NH3 Hydrogen Storage System
Source: Molecules. 2019 Apr 5;24(7):1348. doi: 10.3390/molecules24071348 (PMC6479683; doi:10.3390/molecules24071348)
Supplement: Supplementary file 1 [file molecules-24-01348-s001.pdf]

Supporting information

Eutectic phenomenon of $LiNH_2$-KH composite in MH-NH_3 hydrogen storage system

Kiyotaka Goshome[1], Ankur Jain[2], Hiroki Miyaoka[2], Hikaru Yamamoto[2], Yoshitsugu Kojima[2,3], Takayuki Ichikawa[2,4*]

[1]Renewable Energy Research Center, National Institute of Advanced Industrial Science and Technology, 2-2-9 Machiikedai, Koriyama, Fukushima, 963-0298, Japan

[2]Natural Science Center for Basic Research and Development, Hiroshima University, 1-3-1 Kagamiyama, Higashi-Hiroshima, 739-8530, Japan

[3]Graduate School of Advanced Sciences of Matter, Hiroshima University, 1-3-1 Kagamiyama, Higashi-Hiroshima, 739-8530, Japan

[4]Graduate School of Engineering, Hiroshima University, 1-4-1 Kagamiyama, Higashi-Hiroshima, 739-8527, Japan

* Correspondence: tichi@hiroshima-u.ac.jp (T.I.); ankur.j.ankur@gmail.com(A.J.)

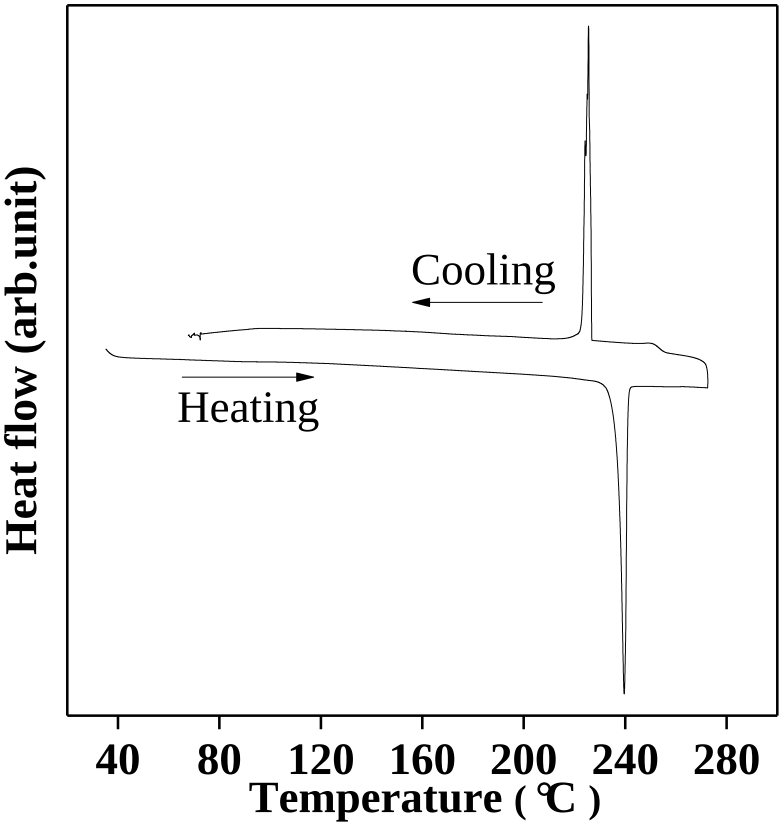

Fig. S1 DSC profile of the reaction product after the hydrogenation of complex $LiK(NH_2)_2$ at 160 ℃. The DSC was performed under 0.5 MPa H_2 atmosphere in closed condition by heating up to 270 ℃ at 5 ℃/min of heating rate.

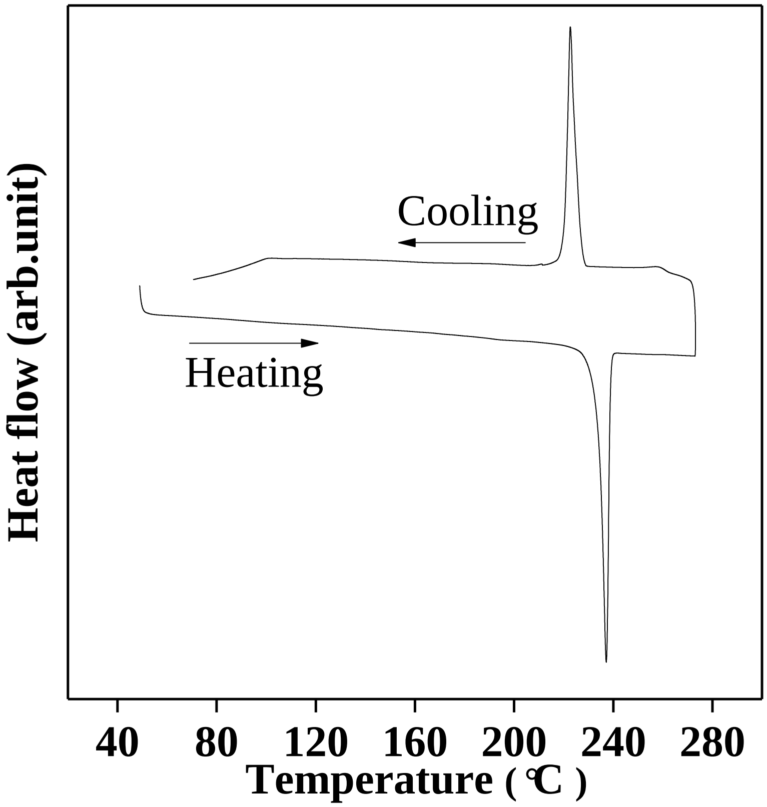

Fig. S2 DSC profile of the ball-milled mixture of $LiNH_2$ and KH. The DSC was performed under 2.0 MPa H_2 atmosphere in closed condition by heating up to 270 °C at 5 °C/min of heating rate.
